# Complete characterization of ultrashort optical pulses with a phase-shifting wedged reversal shearing interferometer

**DOI:** 10.1038/s41377-018-0022-0

**Published:** 2018-07-11

**Authors:** Billy Lam, Chunlei Guo

**Affiliations:** 1 The Institute of Optics, University of Rochester, Rochester, NY 14627 USA; 2 Changchun Institute of Optics, Fine Mechanics and Physics, Chinese Academy of Sciences, 130033 Changchun, China

## Abstract

The ability of an interferometer to characterize the spatial information of a light beam is often limited by the temporal profile of the beam, with femtosecond pulse characterization being particularly challenging. In this study, we developed a simple, stable, controllable shearing and vectorial phase-shifting wedged reversal shearing interferometer that is able to characterize all types of coherent and partially coherent light beams. The proposed interferometer consists of only a single beam splitter cube with one wedged entrance face and is insensitive to environmental vibration due to its common path configuration. A near zero-path length difference of the proposed interferometer ensures its operation for ultrashort pulses, providing, for the first time, a simple and stable interferometric tool to fully characterize sub-100  fs laser pulses. All common beam characterization can be carried out with the interferometer, such as the amplitude, phase, polarization, wavelength, and pulse duration. Furthermore, this technique is sensitive to the wavefront tilt and can be used for precise beam alignment. Therefore, this interferometer can be an essential tool for beam characterization, optical imaging, and the testing required for a wide range of applications, including astronomy, biomedicine, ophthalmology, optical testing and imaging systems, and adaptive optics.

## Ultrafast lasers: beam characterization

Characterizing the spatial properties of ultrashort laser pulses should now be easier thanks to the work of two scientists in the USA and China. Billy Lam and Chunlei Guo have developed an interferometer system that is able to measure the wavefront and spatial profile of sub-100 fs coherent and partially coherent laser pulses. Their wedged reversal shearing interferometer (WRSI) is an extremely compact and robust device based around a single optical element, a 50:50 beam splitter cube which has one of its entrance faces shaped into a wedge. The resulting interferogram generated is imaged by a CCD camera and analyzed to reveal information about the beam. Experiments with 65 fs pulses from an amplified Ti:Sapphire laser system show that the system is able to measure many common and important beam parameters, such as amplitude, phase, polarization, and pulse duration.

## Introduction

Spatial characterization of electromagnetic radiation is essential for nearly all optical imaging and testing, covering a broad range of applications, such as astronomy, biomedicine, ophthalmology, optical testing, imaging systems, and adaptive optics^1–8^. Any optimization of optical beams, such as correcting wavefront distortion and improving image qualities, requires clear information on the spatial beam profile and the wavefront. The temporal profile also plays an important role when characterizing the spatial profile of light beams. For example, interferometric spatial characterization tools face more challenges when characterizing pulsed light beams than when characterizing continuous waves. Particularly, the greatest challenges are when characterizing ultrashort pulses at a femtosecond time scale for two main reasons: low fringe visibility and instability. Coherent ultrashort pulses must temporally overlap to interfere, and non-common path interferometers such as Mach–Zehnder and Michelson configurations are susceptible to small mechanical vibrations and air currents^9–11^.

Currently, the most advanced spatial characterization tools that can fully characterize the wavefront are the non-interferometric type—the Shack–Hartmann wavefront sensor that is commonly used for wavefront sensing, and an interferometric type—the lateral shearing interferometer, where two wavefronts are sheared laterally, which is widely used for optical testing^1, 2^. Although the Shack–Hartmann wavefront sensor can measure femtosecond laser pulses, the transverse resolution is limited by the lenslet size^3^. While the lateral shearing interferometer typically has a better resolution, the measurement of femtosecond laser pulses with this method is challenging due to the extremely short coherence length for interference to occur. Even the common-path cyclic shearing interferometer is only capable of characterizing beams with coherence times down to 300 fs^12^. There is no interferometric system demonstrated that can stably characterize the wavefront and spatial profile of ultrashort pulses with a sub-100 fs pulse duration. As ultrafast femtosecond lasers are widely used in a broad range of research fields, a simple and stable common-path interferometer for characterizing ultrafast light beams is critical.

In this work, we demonstrate a general interferometric approach to spatially characterize any light beam, including femtosecond laser beams. Our approach consists of a single optical element. It is simple, stable, and suitable for a wide range of beam sizes and ultrafast light beams. All common beam parameters can be characterized, including the amplitude, phase, polarization, pulse duration of transform-limited pulses, and wavelength. As a demonstration for this paper, we characterize a femtosecond laser beam with a pulse duration of 65 fs. In fact, our proposed interferometer, the wedged reversal shearing interferometer (WRSI), is capable of characterizing pulses with an arbitrarily short pulse duration. Furthermore, it has the additional capability of measuring the wavefront tilt, unlike common shearing interferometers^2^. Moreover, the WRSI can be rotated to define a unique optical axis while changing the shearing direction. With the unique optical axis and its additional ability to measure the wavefront tilt, this system is used for the first time as a highly sensitive alignment tool to precisely align optics along the optical axis and align multiple laser beams (mutually coherent or incoherent, polarized or unpolarized) to be perfectly collinear. As is known, optical alignment is extremely important in all optical systems. Aligning optics onto the optical axis and laser beams in the designated direction are crucial to the performance of all optical systems.

The paper is organized as follows. We first introduce the set-up and the operation principle of the WRSI in Materials and methods section. A mathematical derivation for the interpretation of the interferogram is provided. Next, experiments are conducted to demonstrate the WRSI.

## Materials and methods

**Fig. 1:**
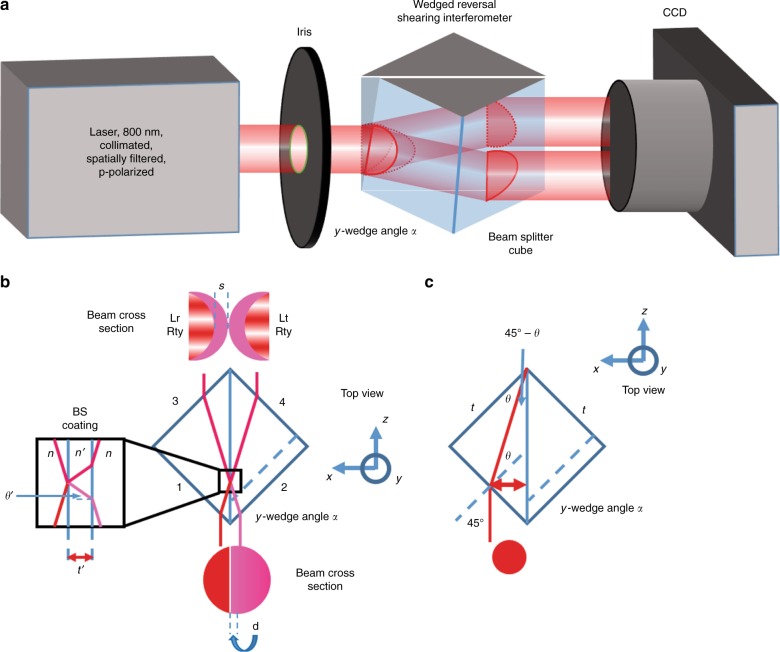
The proposed WRSI for beam characterization. **a** Schematic diagram of the experimental set-up. The *y*-wedge angle is highly exaggerated. **b** Top view of the beam splitter cube that operates as a WRSI. The dashed line shows the cross-section of the BSC at a different height from the base of the BSC. **c** Ray diagram of the WRSI when it is operating with maximum shear with the maximum beam size

Figure 1a presents the schematic illustration of the experimental set-up. The WRSI consists of a single 50:50 beam splitter cube (BSC) with a tiny *y*-wedge angle *α* on one of the entrance faces. The BSC is mounted on a *x*–*y* two-dimensional translation stage on a six-axis kinematic optic mount. To demonstrate that the WRSI can be used for ultrafast light beams, we tested the WRSI using an amplified Ti:Sapphire laser system. The laser system produces 65-fs pulses with a maximum pulse energy of an approximately 1 mJ/pulse at a 1 kHz repetition rate and a central wavelength of 800 nm. The power of the laser can be controlled with an attenuator consisting of a half-wave plate and a polarizer. One should operate the WRSI below the damage threshold of the BSC and the saturation of the charge-coupled device (CCD) camera. The beam is clipped with an iris so that the clipped beam will pass through the WRSI and the aperture of the CCD camera entirely. A CCD imaging lens is used to image the interferogram onto the CCD camera with a de-magnified beam size without diffraction effects due to the beam cutoff.

Figure 1b shows the operation principle of the WRSI. The laser beam splits and incidents onto both faces 1 and 2 of the BSC at an incident angle of 45°. By Snell’s law, the laser beam bends toward normal, and then incidents on the beam-splitting surface with the coating and splits for the second time. Hence, the beam is divided into four parts and then exits through faces 3 and 4 of the BSC. The beams exiting face 3 consists of two parts: the reflected beam from the left portion of the beam having a reversed wavefront and the transmitted beam from the right portion of the beam with a *y*-tilt. The beams exiting face 4 consists of two parts: the transmitted beam from the left portion of the beam and the reflected beam from the right portion of the beam with a *y*-tilt and a reversed wavefront. The *y*-tilt is due to the *y*-wedge angle of face 2, which can be obtained by simply rotating one of the right-angle prisms when the two prisms are assembled together.

Our approach shows some similarities to the Gate’s interferometer, a type of reverse-shearing interferometry, which was presented in a *Nature* paper for optical testing in 1955, before the invention of the laser^13^. However, our work has a range of marked differences. First, Gate’s interferometer characterizes the test surface but not the incident beam^13^. Second, unlike that set-up, we use a wedged prism to obtain phase shifting and fully extract the phase information. As previous works have not provided a detailed theoretical derivation for reverse shearing interferometers^2, 13, 14^, we will present a complete theoretical basis for all types of reverse shearing interferometers, including Gate’s interferometer^13^. Additionally, for the first time, we utilize the reverse shearing interferometry to spatially characterize ultrashort pulses.

If we examine the two beams exiting face 3, the existence of the *y*-tilt results in periodic fringes with a period of [(*n*−*n*_air_)sin*α*]/*λ* for a collimated beam, where *n* and *n*_air_ are the refractive indices of the BSC and air, respectively, and *λ* is the wavelength. Using this equation, one can measure the wavelength if the wedge angle is known and vice versa. The optical path difference (OPD) between the beams is derived in the Supplementary Information and the final result is shown below:

1 $$\phi \left( {x,y} \right) =\, n^{\prime}t^{\prime}\sec \theta^{\prime} + \left( {n - n_{{\mathrm{air}}}} \right)t\left( y \right)\\ - s\frac{{\partial W_{\mathrm{e}}}}{{\partial x}} + s\frac{{\partial W_{\mathrm{o}}}}{{\partial x}} + 2W_{\mathrm{o}}\left( {x,y} \right)$$

where *n*′, *t*′, and *θ*′ are the refractive index, thickness, and the beam refraction angle of the beam splitting coating, respectively; *t*(*y*) = *y*sin*α* ≈ *yα* is the difference in the thickness of the two entrance faces resulting from the *y*-tilt of face 2; and *s* = 2*d* is the shearing amount, wherein *d* is the displacement of the plane of symmetry of the BSC (which can be continuously varied by translating the BSC) from the optical axis of the beam. The function *W*(*x,y*) is the wavefront of the incident beam, which is separated into odd- and even-order terms of the shearing direction *x*: *W*(*x*,*y*) = *W*_e_(*x*,*y*) + *W*_o_(*x*,*y*).

The first term in Eq. (1) describes the contribution of the OPD from the beam splitting coating. This term is comparable to the coating thickness *t*′, which is usually on the order of µm. The second term is a linear function of *y* due to the *y*-wedge angle of face 2. As a result, the second term can cancel out with the first term by translation in the *y* direction. This cancellation ensures that this WRSI can be applied for ultrafast light beams with an ultrashort coherence length. The shearing direction can also be continuously varied by rotating the BSC with respect to the optical axis of the light beam. From Eq. (1), the WRSI measures the wavefront slope (third and fourth terms on the right-hand side) and thus the wavefront. Complete wavefront characterization requires two measurements of the WRSI with orthogonal shearing directions. The odd-order terms of the wavefront, excluding tilt, can be solved by fitting the even-order terms to $s\partial W_{\mathrm{o}}/\partial x$. The even-order terms can then be solved by fitting the odd-order terms to $- s\partial W_{\mathrm{o}}/\partial x + 2W_{\mathrm{o}}\left( {x,y} \right)$. There is an ambiguity between the defocus and tilt for a single measurement. However, the tilt can be eliminated by properly aligning the WRSI. See Supplementary Information for the full procedure of aligning the WRSI.

Although two interferograms with an orthogonal shearing direction contain information for wavefront characterization, the phase retrieval may not be very precise as finding the location of the fringe centers is a difficult task. To extract the phase information from the interferograms, we need to rely on the analytical technique of phase-shifting interferometry (PSI). PSI, as indicated by its name, recovers the phase information from three or more measurements of two beam interferences by inducing a known time-varying phase shift in one beam^2, 15–17^. In our case, we induce the phase shift using a linear translator along the *y* direction. As PSI does not rely on finding the fringe centers and can reconstruct the wavefront point-by-point, it has been the preferred analysis technique for high-precision surface measurements for over 30 years and is still widely used today^18^. The PSI allows the precise measurement of the phase in Eq. (1) and thus the wavefront by curve fitting. The phase retrieval is as follows: four steps of phase shifts, 0, *π*/2, *π* and 3*π*/2, result in four interferograms, and their intensity distributions can be used to extract the phase:

2 $$\phi (x,y) = \arctan \left( {\frac{{I(x,y;3\pi /2) - I(x,y;\pi /2)}}{{I(x,y;0) - I(x,y;\pi )}}} \right)$$

where *I*(*x*,*y*;*δ*) is the intensity distributions of the interferogram with phase shift of *δ*. The full derivation of the amplitude and phase retrieval is provided in Supplementary Information.

One can also operate the WRSI in the other direction with faces 3 and 4 as the entrance faces and faces 1 and 2 as the exit faces. The fringe pattern will depend on the difference between the wedge angles of faces 3 and 4, instead of those of faces 1 and 2. Thus there are two options for an effective wedge angle for a single WRSI.

In addition, this WRSI works for a wide range of beam sizes. The upper limit of the size is limited by the physical size of the BSC. The WRSI works as long as the rays refract and incident on the beam splitting surface. As seen in Fig. 1c, the maximum beam size can be calculated using Snell’s law^2^, where *n*_air_sin45°=*n*sin*θ*. This upper limit is

3 $${\mathrm{Maximum}}\;{\mathrm{beam}}\;{\mathrm{diameter}} = \frac{{t\sin (45^\circ - \theta )}}{{\cos \theta }}$$

where *t* is the side length of the BSC, and $\theta = \arcsin (1/n\sqrt 2 )$ is the refraction angle in the BSC. The maximum beam diameter is approximately equal to 0.33 inches for *t* = 1 inch, *n*_air_ = 1, and *n* = 1.5. The WRSI works for beam sizes smaller than this upper limit but the number of usable fringes will be too few when the beam size is too small. Thus the lower limit of the beam size is limited by the effective wedge angle. Equation (3) can also calculate the lateral displacement of the beam. Thus the spatial chirp of different wavelength components can be calculated with this equation. The spatial chirp experienced by our laser beam with a central wavelength of 800 nm with a 30 nm bandwidth is approximately 5 µm. This will become even smaller on the image plane since the image will be smaller, making this less than the pixel size of the CCD camera. Therefore, we can neglect the effect of the spatial chirp in our pulse. Note that the effect of the spatial chirp is proportional to the bandwidth and the rate of change of the refractive index *dn*/*dω*.

## Results and Discussion

**Fig. 2:**
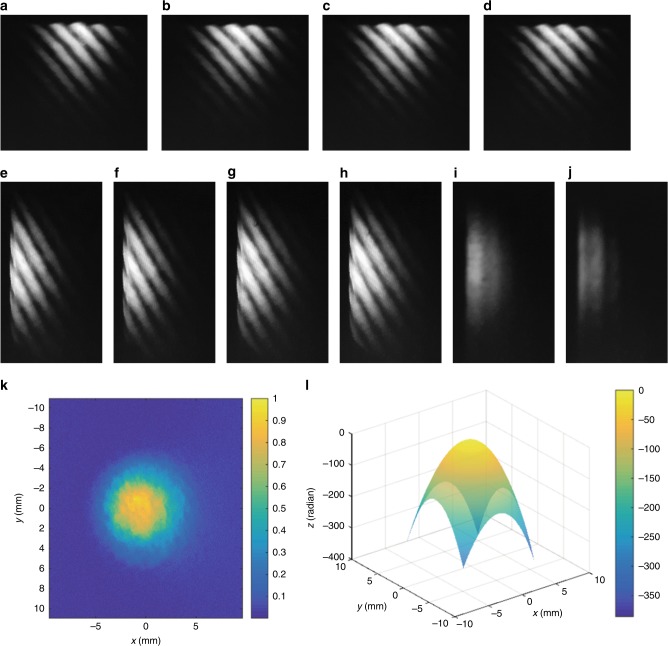
Amplitude and wavefront retrieval of a defocused beam with a pulse duration of 65 fs using the WRSI. **a**–**h** CCD images of the interferograms with a shearing amount of 200 µm for a defocused beam with the BSC translated orthogonal to the shearing direction by distances of 0 $\hat x$ (along the *x* direction), 0.4 mm $\hat x$, 0.8 mm $\hat x$, 1.2 mm $\hat x$, 0 $\hat y$ (along the *y* direction), 0.4 mm $\hat y$, 0.8 mm $\hat y$, and 1.2 mm $\hat y$, respectively. **i**, **j** Beam profiles with faces 1 and 2 of the BSC blocked, respectively. **k** Normalized intensity distribution of the incident beam by combining (**i**) and (**j**). **l** Measured wavefront after phase retrieval using Eq. (2) and curve fitting

First, we characterize the amplitude and phase of a defocused beam with a pulse duration of 65 fs. This is done by producing interferograms with the same shearing amount but in the orthogonal shearing direction. Then we extract the phase and the wavefront of the beam using the analytical technique of PSI. The phase shift of the WRSI is introduced by translating the BSC orthogonal to the shearing direction using a linear translation stage, and then the phase is calculated using Eq. (2). The intensity of the beam is measured by blocking the entrance face one at a time. The related result is shown in Fig. 2. Only half of the interferogram is displayed as the other half contains no additional information. The phase is fitted to Zernike polynomials to the third order. The error in the wavefront retrieval is about *λ*/10 due to the fitting error of the phase. One may also retrieve the wavefront by manipulating the phase using numerical integration and arithmetic to acquire the odd and even components instead of curve fitting for a more accurate retrieval. We can see that the translation of the BSC by Δ*y* = 0.8 mm changes the OPD by approximately *π* for the laser beam with a central wavelength of 800 nm. From Eq. (1), the effective wedge angle is about

4 $${\mathrm{\alpha }} = \arcsin \left[ {\frac{{{\mathrm{\lambda /}}2}}{{({\mathrm{n}} - {\mathrm{n}}_{{\mathrm{air}}}){\mathrm{\Delta y}}}}} \right] = 3.4\,{\rm{arcmin}}$$

Note that one can calculate the wavelength based on this equation if the effective wedge angle is known. The translation required for the PSI is much longer than the ones reported in other PSI methods, which require translation on the order of *λ* using an expensive piezoelectric translator with nanometer precision^19^. In addition, the nonlinearity of the piezoelectric-translator-driven mirror and the instability of the optical system, such as air current and vibration of optics, cause changes in the OPD that lead to errors in measurement^19, 20^. Thus the WRSI is more stable and achieves more precise phase shifting. Note that a similar phase-shifting technique already exists for a shear plate utilizing translation in the wedge direction^16^. However, in that scheme, the phase shifting changes the shearing amount as it is dependent on the thickness of the shear plate^16^. Conversely, the shearing amount in our WRSI is unchanged when the BSC is translated perpendicular to the shearing direction.

This phase-shifting technique also allows one to measure the field autocorrelation and the spectrum of ultrashort pulses. By phase shifting, one can sweep across a range of OPD, and the fringe contrast will weaken as the temporal coherent intensity between the beams becomes weaker. The spectrum can be obtained by the Wiener–Khinchin theorem through Fourier transforming the field autocorrelation^21^. Such a measurement provides the pulse duration of transform-limited pulses. By adding a second-harmonic crystal after the WRSI, we can also obtain the intensity autocorrelation for pulse duration measurements^22^. However, the BSC introduces a temporal distortion because of the group velocity dispersion. For the complete characterization of ultrashort pulses in both the spatial and temporal domains, one may use a reflective configuration of the WRSI to minimize the spatial or spectral chirp, which we plan to investigate in future studies.

In fact, the PSI of the WRSI also allows one to measure the polarization distribution of the beam cross-section point-by-point. This is done by inserting a linear polarizer before the WRSI and measuring the amplitude and phase of the horizontally and vertically polarized beams individually. The polarization distribution is then calculated as a vector sum of the electric field distributions of the horizontally and vertically polarized beams. Thus the WRSI is also useful for characterizing the Full-Poincaré beams, a class of fully correlated optical beams that span the entire surface of the Poincaré sphere^23^.

**Fig. 3:**
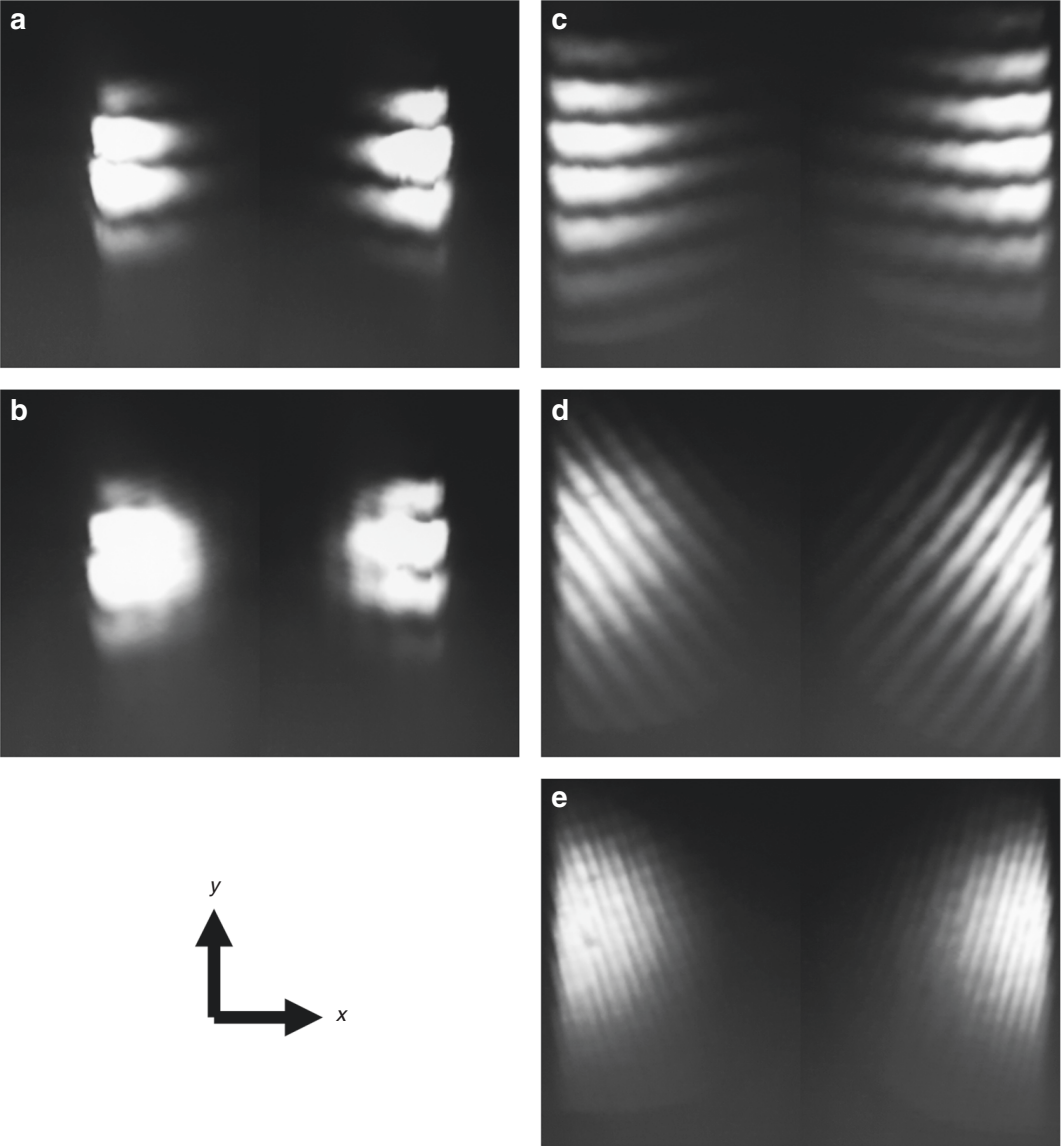
Collimation test with the WRSI. **a**, **b** CCD images of the fringe patterns produced by WRSI of a collimated beam with shearing amounts of *s*_*x*_ = 0 and 2 mm, respectively; **c**–**e** those of a diverging beam with a focal length of *f* = −100 mm with shearing amounts of *s*_*x*_ = 0, 150 µm, and 350 µm, respectively

Next, we demonstrate the WRSI as a quick collimation tester suitable for ultrafast light beams. We insert a diverging lens with focal length of −100 mm into a collimated beam before the WRSI. Figure 3 shows the CCD images of the interferogram for the incident collimated beam and diverging beam at various shearing amounts in the *x* direction. The images with a shearing direction in the *y* direction are shown in Fig. S6 of Supplementary Information. For the collimated beam, the fringe pattern is independent of the shearing amount as shown in Fig. 3a, b. For the diverging beam, varying the shearing amount with a linear translator results in a rotation of the fringes. Note that one does not have to align the WRSI to eliminate the wavefront tilt in order to test the collimation of the beam. The radius of curvature of the wavefront can be calculated by the equation

5 $$R = \frac{{s\Delta }}{{\lambda \tan \gamma }}$$

where Δ is the fringe spacing and *γ* is the orientation angle of the fringes^5^. Using Eq. (5), the focal length is measured to be 102 mm based on the interferogram of Fig. 3d for the diverging beam, which agrees with the focal length provided.

Unlike other shearing interferometers that require a reference mark to indicate the shearing direction^2, 5^, the WRSI has two innate reference marks. As we can see in the interferograms, the pair of fringe patterns rotate in opposite directions for uncollimated beams. Therefore, the pair of fringe patterns become parallel to one another along the shearing direction when the beam is collimated. A similar technique of such self-referencing was also used in the double wedge-plate configuration that requires one to orient the two wedge angles exactly opposite from one another^24, 25^. However, the orientation is susceptible to human error. On the other hand, the WRSI achieves such opposite rotating fringes because the beam exiting faces 3 and 4 are mirror counterparts of each other. This intrinsic symmetry of the system guarantees that the self-referencing of the shearing direction is reliable. Second, the beam is clipped as it passes through the BSC, which acts as another innate reference mark. For any wavefront-symmetric beam, the mirror counterparts of the interferogram are reverse of each other, meaning that the constructive interference in the interferogram corresponds to the destructive interference in the mirror counterpart and vice versa. This is because the two reflected beams are incident onto the beam splitting surface on opposite sides and one of them experiences a *π* phase shift due to reflection from a surface with higher refractive index.

As mentioned earlier, there is an ambiguity between the defocus and tilt in Eq. (1) as they both cause the fringes to rotate. However, these aberrations can easily be distinguished by controlling the shearing amount because the fringe pattern changes with the shearing amount for the defocus but not the *x*-tilt. Therefore, any angular deviation along the *x*-axis can be detected with this WRSI. Similarly, the angular deviation along the *y*-axis can also be detected by rotating the BSC by 90°. The optical axis of the WRSI is uniquely defined as the rotational axis. By Eq. (1), when a beam enters the WRSI with a *x*-tilt of *θ*_*x*_, the interfering beams will have a tilt of 2*θ*_*x*_ with respect to each other. Hence, this WRSI has twice the sensitivity to tilt compared to that of the Michelson interferometer.

**Fig. 4:**
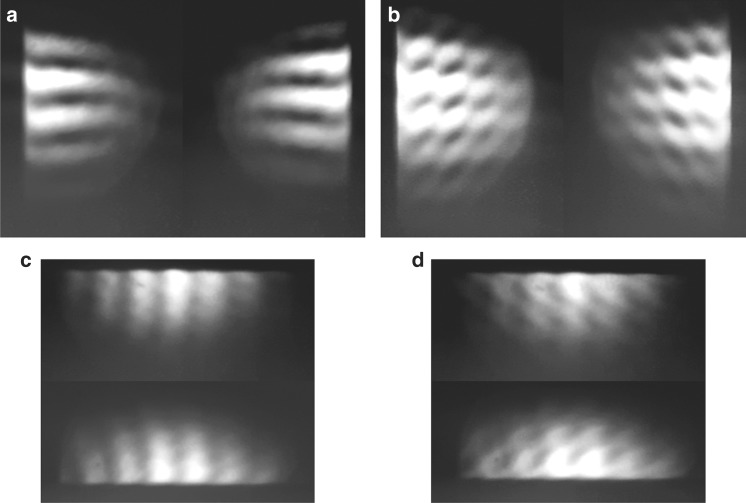
The effect of wavefront tilt on the interferograms of the WRSI. Fringes patterns produced by the WRSI from two beams in a Michelson interferometer where **a** the two beams are collinear with shearing direction in $\hat x$, **b** one beam has *x*-tilt with shearing direction in $\hat x$, **c** the two beams are collinear with shearing direction in $\hat y$, and **d** one beam has a *y*-tilt with shearing direction in $\hat y$

Since the WRSI is sensitive to tilt, we propose this WRSI as a simple and precise alignment tool for aligning multiple collimated collinear beams by simply setting the shearing amount to 0. To test this idea, we generated two collimated beams from a Michelson interferometer and characterized the collinearity of the two propagating beams. The two beams passed through the WRSI to provide an overlay of two interferograms. The propagation direction of one of the beams was tuned with a mirror, and Fig. 4 shows the changes to the overlay of the two interferograms as the mirror was tilted. If the two collimated beams are perfectly collinear, the individual fringe patterns produced by each of the two beams are identical, such as those in Fig. 4a, c. If one of the beams has a tilt in the direction of shear, the fringe pattern of this beam will rotate, and the overlay of the two fringe patterns will be a moiré pattern, as seen in Fig. 4b, d. In a single measurement, the WRSI does not detect wavefront tilt that is orthogonal to the shearing direction. However, the shearing direction can be changed by rotating the BSC to detect both *x*-tilt and *y*-tilt. By tilting the BSC, one can also produce a fringe pattern with a variable periodicity that may find application in Bragg grating fabrication. The technique of overlaying multiple interferograms provides a very effective way to produce a complex moiré pattern that is potentially useful in interference lithography to fabricate complex two-dimensional surface structures.

**Fig. 5:**
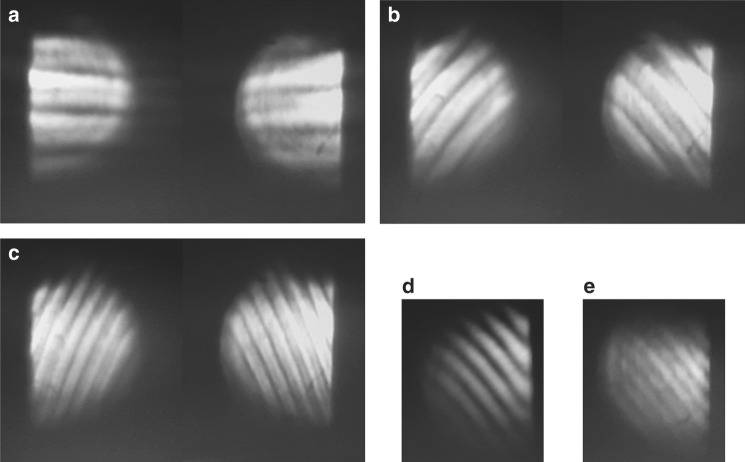
Multiple wavelength characterization by the WRSI. **a**–**c** CCD images of the overlaid fringe patterns from both 400 nm and 800 nm converging beams with a focal length of *f* = 400 mm at shearing amounts of *s* = 0, *s* = 200 µm, and *s* = 400 µm, respectively; **d** that of 800 nm beam at *s* = 200 µm; **e** that of 400 nm beam at *s* = 200 µm

Next, we demonstrate that the WRSI is applicable for a broad spectral range and is not limited to a certain wavelength. In particular, the shearing amount and direction are independent of wavelength. To test the multiple wavelength feature of the WRSI, we insert a beta barium borate (BBO), generating a collinear second-harmonic beam along with the fundamental, followed by a lens with a focal length of *f* = 400 mm and a blue filter before the WRSI. The BBO crystal is misaligned such that the second-harmonic generation efficiency balances out with the attenuation of the fundamental from the blue filter so that the fringe patterns from both wavelengths can be observed simultaneously. The same shearing amount for different wavelengths implies that the orientation angles of the individual fringe patterns are the same. This is confirmed in Fig. 5, wherein two collinear converging beams with wavelengths of 800 nm and 400 nm pass through the WRSI at various shearing amounts. Note that the interferogram of the wavelength component of 400 nm has a lower visibility. This is because the intensity mismatch occurs between the interfering beams caused by a misaligned BBO crystal, and 400 nm does not match the working wavelength of the BSC. A discussion on the visibility of the interference pattern is included in Supplementary Information. With the multiple wavelength feature, one can use white light to also sweep across the different phase shift and find the zero-order fringe, and thus the zero-path length difference.

**Fig. 6:**
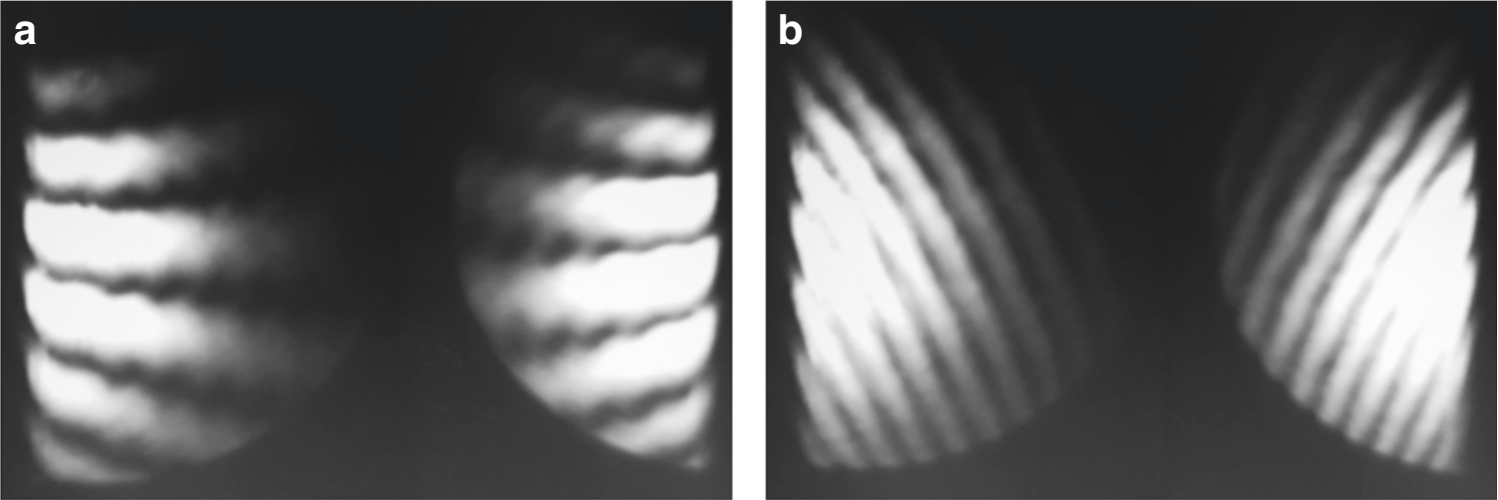
Len alignment by the WRSI. **a**, **b** Fringe patterns produced by the WRSI for a collimated beam that passed through a diverging lens with an oblique angle of 20° at a shearing amount of *s* = 0 and 150 µm, respectively

After proper alignment, the WRSI can be used as an alignment tool for aligning optics (such as converging or diverging lenses) along the optical axis. For example, when focusing or defocusing a beam, the alignment of a lens is important: the center of the lens should coincide with the center of the laser beam, and the laser beam should be normal incident to the lens. Here we demonstrate how to precisely control this alignment with our WRSI. The lenses should be aligned such that the fringe patterns are parallel to the shearing direction with zero shear, as shown in Fig. 3c and S6c. This guarantees that the propagation direction is unchanged. Figure 3 and S6 show the fringe patterns produced by the WRSI of a well-aligned diverging lens at various shearing amounts and directions. If a lens is passed under an oblique angle or a plano-convex lens is inserted in the wrong orientation, this will lead to higher-order aberrations that manifest themselves as curved fringes generated by the shearing interferometer^26^. Moreover, the reverse wavefront mechanism of the WRSI provides the feature of testing the symmetry of the beam in terms of its intensity as well as wavefront. The asymmetry of the intensity profile of the beam will give rise to a lower fringe visibility depending on the intensity ratio. On the other hand, the asymmetry of the wavefront will result in curved fringes in the interferogram of the WRSI even at a shearing amount of *s* = 0 as seen in Fig. 6a. This asymmetry is the highest-order term $2W_{\mathrm{o}}(x,y)$ in Eq. (1). Thus it has a much higher sensitivity to the odd-order terms than do typical shearing interferometers^2^. The oblique angle misalignment of the lens causes the fringe pattern to change; the further away from the center along the shearing direction, the greater the deviation. When the lens is well aligned, the fringe pattern produced by the WRSI is primarily due to a spherical aberration present in the lens.

Lastly, we will show that the fringe visibility depends on beam polarization. When the incident polarization is not vertically or horizontally polarized, the fringe contrast will be reduced. This effect can be described mathematically using the Jones vector formalism. The Jones matrices and vectors used in our mathematical description can be found in ref. ^27^. The Jones vector representing an incident beam with an arbitrary polarization is

6 $${\mathrm{j}}_{{\mathrm{in}}} = \left( {\begin{array}{*{20}{c}} {\cos {\mathrm{\beta }}} \\ {{\mathrm{e}}^{{\mathrm{i}}\varphi }\,\sin {\mathrm{\beta }}} \end{array}} \right)$$

In the WRSI, the incident beam is partially reflected and partially transmitted and recombined to interfere. The Jones vectors of the two beams exiting face 3 of the BSC can be written as

7 $${j}_{\mathrm{t}} = \left( {\begin{array}{*{20}{l}} {{t}_{\mathrm{p}}} \hfill & 0 \hfill \\ 0 \hfill & {{t}_{\mathrm{s}}} \hfill \end{array}} \right){j}_{{\mathrm{in}}} = {t}\left( \begin{array}{l}\cos \,\beta \\ {\mathrm{e}}^{{\mathrm{i}}\varphi }{\mathrm{sin}}\,\beta \end{array} \right)$$

8 $$j_{\mathrm{r}} = \left( {\begin{array}{*{20}{c}} { - r_{\mathrm{p}}} & 0 \\ 0 & {r_{\mathrm{s}}} \end{array}} \right)j_{{\mathrm{in}}} = r\left( \begin{array}{l} - {\mathrm{cos}}\,{\mathrm{\beta }}\\ {\mathrm{e}}^{{\mathrm{i}}\varphi }{\mathrm{sin}}\,{\mathrm{\beta }}\end{array} \right)$$

where *t*_p_=*t*_s_=*t*=*r*_p_=*r*_s_=*r* is the transmission and reflection coefficient of p-polarized light and s-polarized light. These are assumed to be equal for simplicity. Two beams with the same polarizations interfere, and the intensity is dependent on the OPD. On the other hand, two beams with orthogonal polarizations superimpose to create a polarization gradient that is dependent on the OPD. By decomposing the Jones vector into the projected and orthogonal components, we can determine the fringe contrast:

9 $$\left( {\begin{array}{*{20}{c}} { - \cos \beta } \\ {e^{{\mathrm{i}}\varphi}\sin \beta } \end{array}} \right) = - \cos 2\beta \left( {\begin{array}{*{20}{c}} {\cos \beta } \\ {e^{{\mathrm{i}}\varphi }\sin \beta } \end{array}} \right) \\ + \sin 2\beta \left( {\begin{array}{*{20}{c}} { - \sin \beta } \\ {e^{{\mathrm{i}}\varphi }\cos \beta } \end{array}} \right)$$

**Fig. 7:**
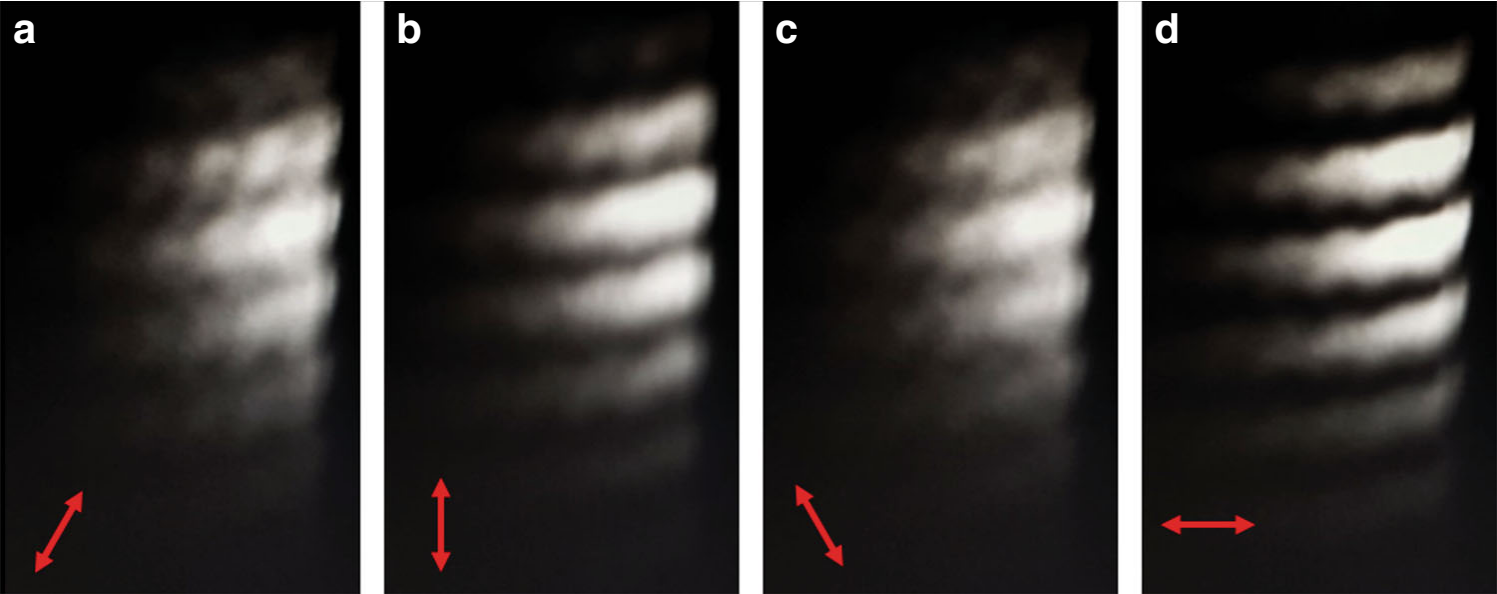
**Fringe visibility study of the WRSI. a**–**d** CCD images of the fringe patterns produced by the WRSI with HWP at angles of 30°, 45°, 60°, and 90°, respectively. The arrow indicates the incident polarization

The fringe contrast is then $|\cos 2\beta |$. Therefore, we can continuously vary the fringe contrast by inserting a half-wave plate before the WRSI to rotate the linear polarization. Figure 7 presents the fringe pattern produced by the WRSI with a different incident beam polarization. The fringe visibility is maximized when the incident polarization is horizontal or vertical polarization and is minimized when the incident beam is 45° polarized. By Eqs. (7) and (8), the negative sign in front of *r*_p_ causes a phase shift of *π* for the p-polarized beam but no phase shift for the s-polarized beam. Therefore, the fringe pattern will alternate between constructively and destructively interfering. For an ideal 50/50 BSC, the WRSI is energy efficient as the four output beams of the WRSI have the same total power as the incident beam.

## Conclusion

We have conceptualized and developed a WRSI that is applicable for ultrafast light beams and suitable for a wide range of beam sizes. It can fully characterize all the common parameters of an optical beam, including amplitude, phase, polarization, pulse duration of transform-limited pulses, and wavelength. The device is stable and insensitive to environmental vibration due to its common-path configuration. The WRSI is very simple and compact because it consists of only a single BSC, and it has a high energy efficiency and a high fringe contrast. Owing to its simplicity, the whole system can be easily translated and/or rotated to obtain continuous variable shear, shearing direction, and phase shifting as well as beam alignment because WRSI is sensitive to wavefront tilt. This interferometer can be an essential tool for optical imaging and testing, which are required for a wide range of applications, including astronomy, biomedicine, ophthalmology, optical testing and imaging systems, and adaptive optics.

## Electronic supplementary material

Supplementary material
