# Supplementary material for: Complete characterization of ultrashort optical pulses with a phase-shifting wedged reversal shearing interferometer
Source: Light Sci Appl. 2018 Jul 11;7:30. doi: 10.1038/s41377-018-0022-0 (PMC6106999; doi:10.1038/s41377-018-0022-0)
Supplement: Supplementary file 1 — Supplementary material [file 41377_2018_22_MOESM1_ESM.pdf]

Complete characterization of ultrashort optical pulses with a phase-shifting wedged reversal shearing interferometer: supplementary information

Billy Lam[1,2] and Chunlei Guo[1,2,a)]
[1] *The Institute of Optics, University of Rochester, Rochester, New York, 14627, USA and*
[2] *Changchun Institute of Optics, Fine Mechanics and Physics, Changchun 130033 China*

(Dated: 15 May 2018)

This document provides supplementary information to "Complete characterization of ultrashort optical pulses with a phase-shifting wedged reversal shearing interferometer." This document provides the theoretical derivation for the operation of the reversal shearing interferometer, discusses amplitude and phase retrieval of the phase-shifting interferometry (PSI), outlines the procedure of aligning the Wedged Reversal Shearing Interferometer (WRSI) perfectly for the vectorial shearing feature and provides the interferograms with different shearing directions.

I. OPERATION PRINCIPLE OF WEDGE REVERSAL SHEARING INTERFEROMETER (WRSI)

Figure S1 shows the operation principle of the WRSI. The beams exiting face 3 consist of two parts: the reflected beam from the left portion of the beam with reversed wavefront and the transmitted beam from the right portion of the beam with y-tilt. The reflected and transmitted beams pass through nearly equal thickness of the beam splitter cube (BSC). However, the transmitted beam has to propagate through the BS coating between the two prisms while the reflected one does not. So the optical path difference (OPD) between the beams is

$$OPD \approx n't' \sec\theta' + (n - n_{\text{air}})t(y) + W(x,y) - W(-x-s,y) \qquad \text{(S1)}$$

where n', t' and θ' are the refractive index, thickness and the beam refraction angle of the coating respectively, $t(y) = y\sin\alpha \approx y\alpha$ is the thickness difference of the two entrance faces resulted from y-tilt of face 2, and $s = 2d$ is the shearing amount with d being the displacement of plane of symmetry of the BSC (which can be continuously varied by translating the BSC) from the optical axis of the beam. The function $W(x,y)$ is the wavefront of the incident beam, which can be separated into odd and even order terms and then the terms containing the wavefront become $W_{\text{e}}(x,y) - W_{\text{e}}(x+s,y) + W_{\text{o}}(x,y) + W_{\text{o}}(x+s,y)$. To simplify the terms, we taylor expand the sheared wavefront as follows:

$$W(x+s,y) = W(x,y) + s\frac{\partial W(x,y)}{\partial x} + \frac{s^2}{2!}\frac{\partial^2 W(x,y)}{\partial x^2} + \mathcal{O}(s^3). \qquad \text{(S2)}$$

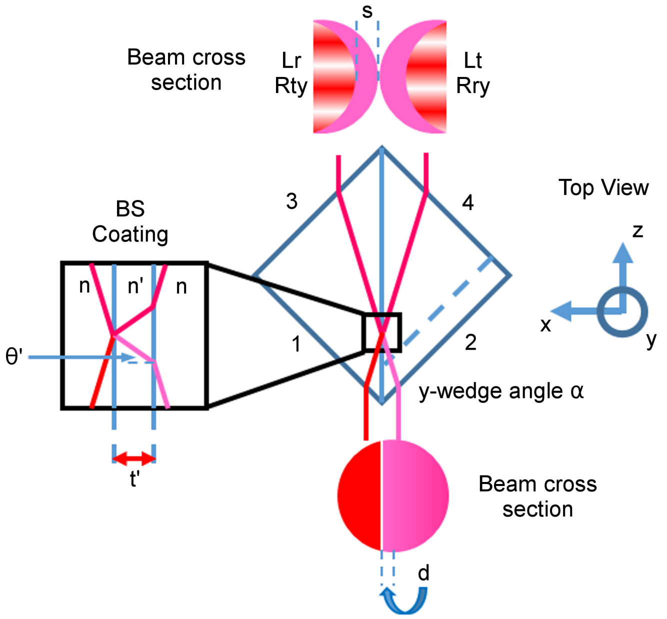

FIG. S1. The beam splitter cube that operates as a WRSI.

The shearing amount s is chosen to be small for wavefront retrieval, so we keep only the two highest order terms of s as such:

$$W(x,y) - W(-x-s,y) = 2W_{\text{o}}(x,y) + s\frac{\partial W_{\text{o}}}{\partial x} - s\frac{\partial W_{\text{e}}}{\partial x}. \qquad \text{(S3)}$$

Plugging this back into the OPD Eq. (S1) yields

$$\phi(x,y) \approx n't' \sec\theta' + (n - n_{\text{air}})t(y) - s\frac{\partial W_{\text{e}}}{\partial x} + s\frac{\partial W_{\text{o}}}{\partial x} + 2W_{\text{o}}(x,y). \qquad \text{(S4)}$$

a) Electronic mail: guo@optics.rochester.edu

II. AMPLITUDE AND PHASE RETRIEVAL BY PSI

For the Wedged Reversal Shearing Interferometer, four steps of phase shift $0, \pi/2, \pi, 3\pi/2$ result in four interferograms that can be written as the following intensity distributions:

$$I(x,y;0) = I'(x,y) + I''(x,y)\cos(\phi(x,y)) \quad \text{(S5)}$$
$$I(x,y;\pi/2) = I'(x,y) - I''(x,y)\sin(\phi(x,y)) \quad \text{(S6)}$$
$$I(x,y;\pi) = I'(x,y) - I''(x,y)\cos(\phi(x,y)) \quad \text{(S7)}$$
$$I(x,y;3\pi/2) = I'(x,y) + I''(x,y)\sin(\phi(x,y)) \quad \text{(S8)}$$

where $I'(x,y) = I_1(x,y) + I_1(-x-s,y)$ is the sum of the intensity of the two interfering beams, $I''(x,y) = 2\sqrt{I_1(x,y)I_1(-x-s,y)}$. Solving for I', I'', and ϕ yields

$$I'(x,y) = (I(x,y;0) + I(x,y;\pi))/2 \quad \text{(S9)}$$
$$I''(x,y) = (I(x,y;0) - I(x,y;\pi))/(2\cos(\psi(x,y))) \quad \text{(S10)}$$
$$\phi(x,y) = \tan^{-1}\left(\frac{I(x,y;3\pi/2) - I(x,y;\pi/2)}{I(x,y;0) - I(x,y;\pi)}\right) \quad \text{(S11)}$$

The amplitude of the incident beam $I_1(x,y)$ can be solved using the quadratic formula on the following the quadratic equation because I' is the sum of roots and $I''^2/4$ is the product of roots:

$$0 = I_1^2 - I'I_1 + I''^2/4 \quad \text{(S12)}$$
$$I_1(x,y) = \frac{I' \pm \sqrt{I'^2 - I''^2}}{2} \quad \text{(S13)}$$

There is a sign ambiguity for the intensity $I_1(x,y)$ because the opposite sign corresponds to $I_1(-x-s)$. The sign ambiguity can be resolved with a measurement of the beam intensity by blocking one entrance face of the beam splitter cube.

III. SIMPLER AMPLITUDE AND PHASE RETRIEVAL METHOD

As mentioned before, one can simply measure the intensity distribution by blocking one of the entrance faces of the BSC and measure $I_1(x,y)$ and $I_1(-x-s)$ individually. Note that one can also measure $I_1(x,y)$ without the WRSI. If $I_1(x,y)$ and $I_1(-x-s)$ are known, the phase can be solved with a single interferogram by

$$\phi(x,y) = \cos^{-1}\left(\frac{I(x,y;0) - I'(x,y)}{I''(x,y)}\right) \quad \text{(S14)}$$

IV. VISIBILITY OF THE INTERFERENCE

From the two beam interference Eq. (S5), the visibility is simply $I''(x,y)/I(x,y) = 2\sqrt{I_1(x,y)I_1(-x-s,y)}/(I_1(x,y) + I_1(-x-s,y))$. The visibility equals to unity for a symmetric beam with shearing amount of $s = 0$ m. Any intensity mismatch will cause visibility to decrease.

V. GROUP VELOCITY DISPERSION

Group velocity dispersion (GVD) causes chirping for both the reflected and transmitted beam of the BSC. The GVD is equal to $\frac{dv_{\mathrm{g}}^{-1}}{d\omega}$ where v_{g} is the group velocity. The GVD of the material BK7 at the wavelength of 800nm is 44.65 fs^2 mm^{-1} according to Schott glass catalog. This BSC has a positive dispersion and causes a positive chirp (higher frequency lags behind). Using linear approximation, the group velocity mismatch between 785 nm and 815 nm (our laser bandwidth is 30 nm) is 3.95 fs mm^{-1}. Therefore, a positively/negatively chirped pulse is broadened/shortened by toughly 4 fs for every millimeter it propagates through the BSC. Note that this chirping effect is proportional to the GVD, bandwidth, and the dimension of the BSC. As a comparison, the group velocity mismatch of a BBO crystal for type I second harmonic generation at 780 nm is 280 fs mm^{-1} [1]. Therefore, our 1-inch cube will induce about the same chirping as a 0.3-mm BBO crystal.

VI. BSC SIZE AND GVM TRADEOFF

Larger BSC sizes allows for characterization of larger beam sizes. However, the induced chirp by the BSC is also proportional to the size of the BSC as discussed in the previous section. This would reduce the accuracy of the temporal measurement. To keep this reduction of accuracy in check, the following criteria should be followed: $(GVM)L < \tau_c$, where L is the length of the BSC and τ_c is the pulse coherence time. Therefore, shorter pulses should be characterized using a smaller BSC if the sizes permits.

In the future, we plan to investigate and completely resolve the issue of temporal distortion for the temporal measurement using WRSI and autocorrelation or other temporal measurement techniques. We would like to tackle this issue using the following viable options: building a reflective variant of the WRSI, or correcting GVM errors by accurately measuring the difference in induced GVM between using BSC with two distinct sizes.

VII. ALIGNMENT PROCEDURE FOR WRSI

In order to use this Wedged Reversal Shearing Interferometer (WRSI) as an alignment tool for aligning lenses, we must first align the WRSI onto the optical axis. To do so perfectly, we must align the hypotenuse face of the beam splitter cube (BSC) onto the rotation axis and the rotation axis onto the optical axis without any shearing

nor tilt in both the x and y direction. Initially, a collimated beam propagating along the optical axis is needed. Note that this WRSI can test the collimation simply by translating the BSC. The BSC should be mounted on translation stages controlling the shearing amount on a 6-axis kinematic optic controlling the shearing direction by rotation with fine adjustment of the position and the tilt of the rotation axis. The procedure for aligning the WRSI perfectly is separated into two main parts: eliminating tilt and shearing amount. This is easily achieved by exploiting the symmetry of the system.

First, we align the BSC onto the optical axis of the collimated beam with shearing direction in the x-axis. Fringes parallel to the shearing direction can be seen. However, initially the rotation axis will have x-tilt of θ_x with respect to both the optical axis and the hypotenuse face of the BSC as shown in Fig. S2. Rotate the BSC by 180° with respect to the rotation axis and then the BSC will have x-tilt of $2\theta_x$. Now, we can correct the x-tilt by rotating the BSC about the y-axis by $-\theta_x$ onto the rotation axis. Closely monitor the fringe pattern, the x-axis spatial frequency (f_X) should be reduced by half. Adjust the x-tilt of the 6-axis kinematic optic mount to eliminate the remaining x-tilt. Fringes parallel to the x-axis should be observed. An example of this is shown in Fig. S3. Next, we eliminate the y-tilt by changing the shearing direction to y-axis and adjust the y-tilt of the 6-axis mount until fringes are parallel to the y-axis.

With the WRSI perfectly parallel to the optical axis, we will eliminate the shearing amounts. To do so, we insert a lens to introduce defocus because the fringe patterns will be dependent on shearing amount. Align the lens such that fringes are parallel to the shearing direction (y-axis). Change the shearing direction to x-axis and do the same. This means that the x-tilt and the y-tilt introduced by the lens balances out with the defocus term in the WRSI. An exaggerated example of this can be seen in Fig. S4 where a large amount of shear balances out with the x-tilt. Again, we exploit the symmetry of the system and rotate the BSC by 180° with respect to the rotation axis. This time, the fringe pattern changes because the shearing amount changes by Δs_x as seen in Fig. S5. Translate the BSC by $-\Delta s_x/2$ so the rotation axis lies onto the hypotenuse face of the BSC. Closely monitor the fringe pattern, the x-axis spatial frequency (f_X) should be reduced by half. The interferogram will resemble Fig. S3. Because of the way we align the lens, tilt and shearing amount coexist and they are directly proportional to each other. Translating the BSC along the rotational axis will change the shearing amount due to tilt and fringes will rotate. We can eliminate the shearing amount by realigning the lens and correcting the shearing amount according to the rate at which fringes rotate from the axial translation.

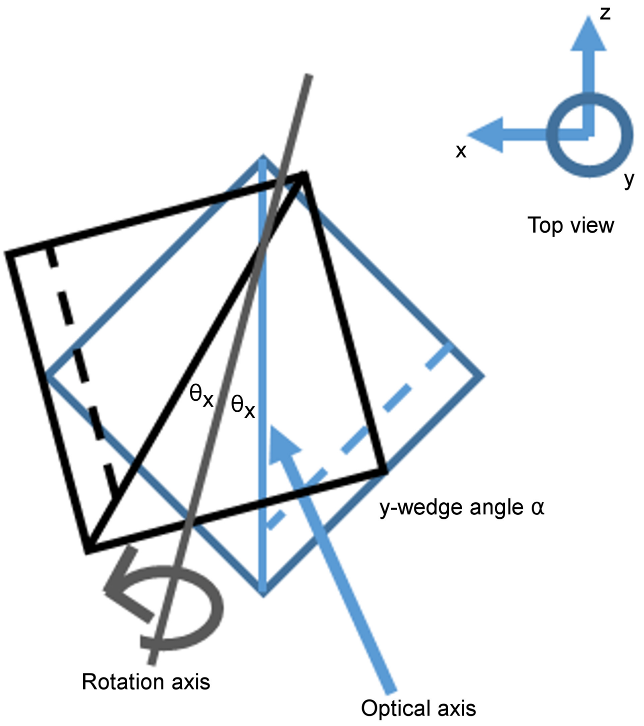

FIG. S2. Top view of BSC on the optical axis and after a 180° rotation about the rotation axis. The rotation axis has x-tilt of θ_x.

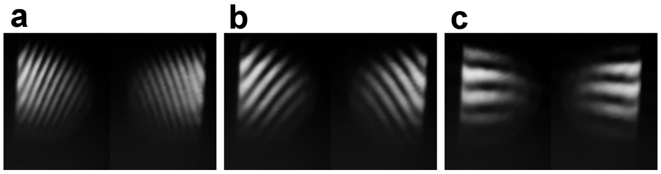

FIG. S3. CCD image of the interferogram produced by the WRSI (**a**) operating with x-tilt θ_x, (**b**) after correcting half of the x-tilt, (**c**) after correcting the remaining x-tilt.

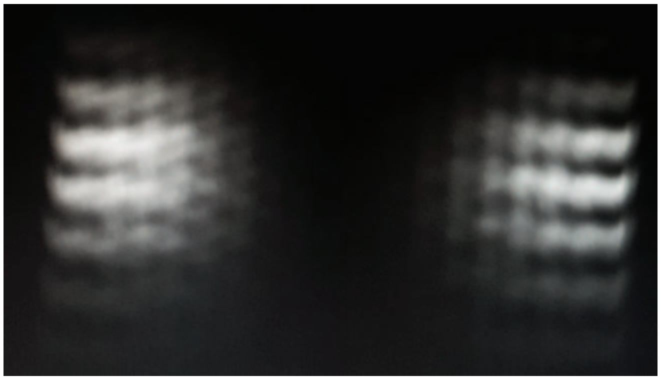

FIG. S4. Fringe pattern produced by a defocused beam with a large shearing amount that balances with the x-tilt.

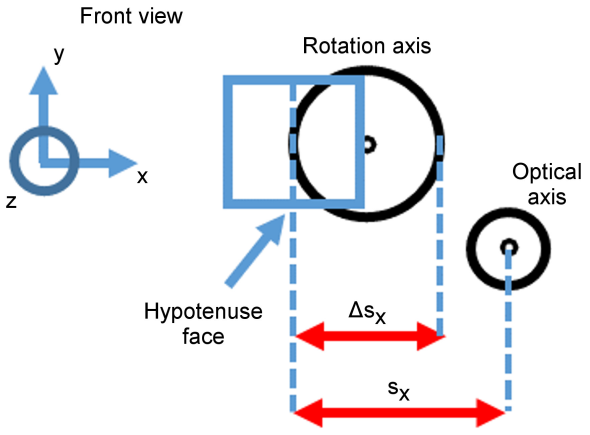

FIG. S5. Front view of BSC with shearing amounts in both x and y axis.

VIII. WRSI WITH SHEARING DIRECTION IN Y-AXIS

Having aligned the WRSI, we tested the vectorial shearing feature of the WRSI using a diverging lens with focal length of -100 mm. Figure S6 shows the CCD images of the sheared beam produced by WRSI for an incident collimated beam and diverging beam at various shearing amounts with the shearing direction in y direction. The same images with shearing directions in the x direction are shown in Fig. 3 in the main paper.

REFERENCES

[1] Krylov V, Rebane A, Kalintsev AG, Schwoerer H, Wild UP. Second-harmonic generation of amplified femtosecond Ti: sapphire laser pulses. Optics letters. 1995 Jan 15;20(2):198-200.

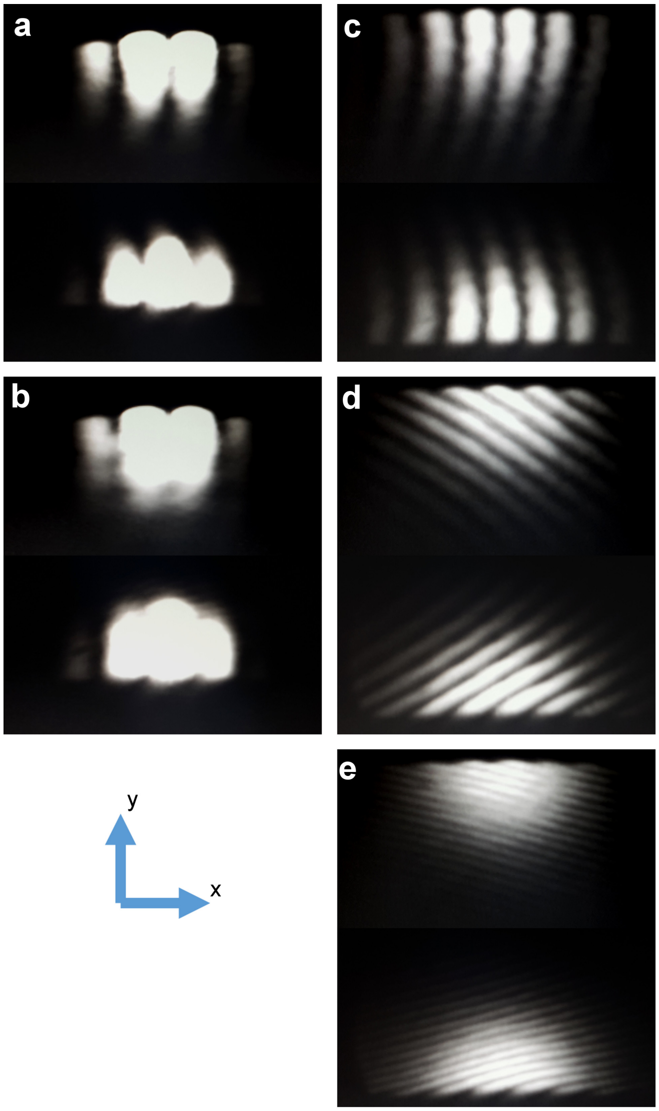

FIG. S6. (**a**-**b**) CCD images of the fringe patterns produced by WRSI of a collimated beam with a shearing amount of $s_y = 0$, and $s_y = 2$ mm, respectively. (**c**-**e**) Those of a diverging beam with $f = -100$ mm with shearing amount of $s_y = 0$, 150 μm, and 350 μm, respectively.
